# A molecular understanding of d-homoestrone-induced G2/M cell cycle arrest in HeLa human cervical carcinoma cells

**DOI:** 10.1111/jcmm.12587

**Published:** 2015-07-31

**Authors:** Renáta Minorics, Noémi Bózsity, Judit Molnár, János Wölfling, Erzsébet Mernyák, Gyula Schneider, Imre Ocsovszki, István Zupkó

**Affiliations:** a Department of Pharmacodynamics and Biopharmacy, University of SzegedSzeged, Hungary; b Department of Organic Chemistry, University of SzegedSzeged, Hungary; c Department of Biochemistry, University of SzegedSzeged, Hungary

**Keywords:** d-homoestrone, tubulin polymerization, stathmin, loss of function of Cdk1, cell cycle arrest, HeLa cells, G2/M phase transition, Cdc25B and C, intrinsic apoptotic pathway

## Abstract

2-Methoxyestradiol (ME), one of the most widely investigated A-ring-modified metabolites of estrone, exerts significant anticancer activity on numerous cancer cell lines. Its pharmacological actions, including cell cycle arrest, microtubule disruption and pro-apoptotic activity, have already been described in detail. The currently tested d-ring-modified analogue of estrone, d-homoestrone, selectively inhibits cervical cancer cell proliferation and induces a G2/M phase cell cycle blockade, resulting in the development of apoptosis. The question arose of whether the difference in the chemical structures of these analogues can influence the mechanism of anticancer action. The aim of the present study was therefore to elucidate the molecular contributors of intracellular processes induced by d-homoestrone in HeLa cells. Apoptosis triggered by d-homoestrone develops through activation of the intrinsic pathway, as demonstrated by determination of the activities of caspase-8 and -9. It was revealed that d-homoestrone-treated HeLa cells are not able to enter mitosis because the cyclin-dependent kinase 1-cyclin B complex loses its activity, resulting in the decreased inactivation of stathmin and a concomitant disturbance of microtubule formation. However, unlike 2-ME, d-homoestrone does not exert a direct effect on tubulin polymerization. These results led to the conclusion that the d-homoestrone-triggered intracellular processes resulting in a cell cycle arrest and apoptosis in HeLa cells differ from those in the case of 2-ME. This may be regarded as an alternative mechanism of action among steroidal anticancer compounds.

## Introduction

The global burden of cancer is continuing to rise, largely because of the ageing and growth of the world’s population and the increasing adoption of cancer-causing behaviour, such as smoking and physical inactivity, within economically developing countries. Cancer is the second leading cause of death both worldwide and in economically developed countries 1. Among women, all types of tumours that affect the reproductive organs (breast, uterus, cervix and ovaries) are to be found in the list of the 10 most frequently diagnosed cancers 2. Cervical cancer is the second most commonly diagnosed cancer, affecting the reproductive system in females throughout the world. It accounted for some 8% of the total new cancer cases and 7.5% of the total cancer deaths among women in 2012 3. Among the economically developed countries, it demonstrates the highest incidence rates in Central and Eastern Europe.

Since 1977, when zur Hausen published his results, it has been known that human papillomavirus (HPV) infection is a fundamental causative factor of cervical cancer 4. Despite the introduction of effective vaccination against this infection, it has emerged that it does not have any therapeutic effect against already-established infections or in asymptomatic carriers 5. The therapeutic interventions include radical surgery, brachytherapy and in most cases platinum-based chemotherapy or a combination of these 6. As concerns the therapeutic difficulties involved in the treatment regimens, adverse effects including haematological and gastrointestinal toxicities or the lack of a significant improvement in survival rate have been reported in connection with cisplatin, in spite of promising drug and/or radiation combinations 7. The need for newer, more potent and better-tolerated drugs for the therapy of this special type of cancer has therefore not declined.

An endogenous metabolite of 17β-estradiol, 2-methoxyestradiol (2-ME), was earlier one of the most widely investigated steroidal anticancer agents, thanks to its potent antiproliferative activity against numerous cancer cell lines [reviewed in 8], including cervical cancer 9–11. Concomitantly, various analogues of 2-ME have been synthetized and tested on cancer cell lines [reviewed in 12], and certain A- and/or d-ring-modified 2-ME derivatives have been reported to exhibit the same or even higher antiproliferative activities (determined most frequently on breast cancer cell lines) than those of 2-ME. However, in the vast majority of the cases, the spectrum of investigations focusing on the mechanisms of action of these potent antiproliferative compounds has been limited to the determination of their effects on tubulin polymerization. It has been concluded that the investigated 2-ME derivatives, increase tubulin depolymerization directly similarly to 2-ME. Until recently, only one research group had published experimental results relating to the mechanism of antiproliferative action of novel 2-ME analogues in HeLa cells 13. The three sulphamoylated analogues investigated induce programmed cell death in HeLa cells *via* the extrinsic and intrinsic apoptotic pathways followed by autophagy. Their action on tubulin polymerization was elucidated through the use of direct, fluorescence-based tubulin polymerization assays and the microscopic analysis of intracellular microtubules. It was revealed that, similarly to 2-ME, the sulphamoylated analogues increase tubulin depolymerization both in a cell-free system and in living cells. These effects were also demonstrated in MDA-MB-231 breast cancer cells treated with the sulphamoylated analogues.

The present test compound, d-homoestrone, is an analogue of 2-ME with structural modifications in its A- and d-rings. This compound was earlier reported to exert potent antiproliferative activity in human cervical cancer cells (HeLa), inducing a cell cycle blockade followed by apoptosis, as demonstrated by morphological markers and caspase 3 activation 14. In consequence of its selective proliferation-inhibiting effect and its structural difference relative to the previously investigated 2-ME analogues, the aim of the present study was to establish whether the intracellular events induced by d-homoestrone in HeLa cells are comparable to those in the case of 2-ME or not. Among others, specific, immune reaction-based flow cytometric analysis, analysis of the mRNA and protein expression of factors involved in the G2/M phase transition and *in vitro* direct tubulin polymerization assays were performed to shed light on this intriguing question.

## Materials and methods

### Chemicals

**Figure 1:**
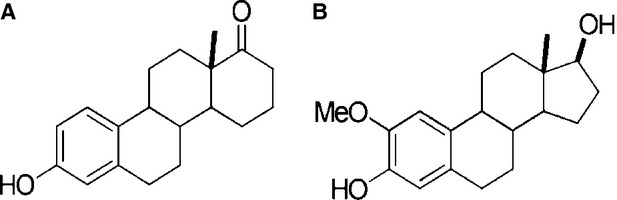
Chemical structures of d-homoestrone (A) and 2-methoxyestradiol (B).

Normal d-homoestrone (Fig. 1) was synthetized by Wölfling *et al*. as described previously 15. All other chemicals and kits, if otherwise not specified, were purchased from Sigma-Aldrich Ltd. (Budapest, Hungary).

### Cell cultures

The HeLa human cervix epitheloid carcinoma cell line was purchased from ECACC (European Collection of Cell Cultures, Salisbury, UK). HeLa cells were grown in DMEM supplemented with 10% heat-inactivated foetal calf serum, 1% non-essential amino acids and 1% penicillin–streptomycin in a humidified atmosphere containing 5% CO_2_ at 37°C. The medium and supplements were obtained from Life Technologies (Paisley, UK). Cell counts were determined with a Z1 Coulter Particle Counter (Beckman Coulter Hungary Ltd., Budapest, Hungary). Cells in the near-confluent phase of growth were used in all the studies described below.

### Antiproliferative (MTT) assay

The growth-inhibitory effects of d-homoestrone were determined on SiHa (an HPV-16-positive cervical cancer cell line) and C33A (an HPV-negative cervical cancer cell line) cells to demonstrate the selectivity of its antiproliferative effect towards HPV-18-positive cervical cancer cells. Experiments were carried out in the same way as in the previously described investigations on HeLa cells 14. Briefly, all cell types were seeded into 96-well plates at a density of 5000 cells/well and incubated with increasing concentrations (0.1–30 μM) of d-homoestrone at 37°C under cell culturing conditions. After a 72-hrs incubation, cells were treated with 5.0 mg/ml MTT (3-(4,5-dimethylthiazol-2-yl)-2,5-diphenyltetrazolium bromide) solution for 4 hrs, the precipitated formazan crystals were dissolved in dimethyl sulphoxide, and the absorbance was read at 545 nm with a microplate reader; wells with untreated cells were utilized as controls 16. Sigmoidal dose–response curves were fitted to the measured points, and the IC_50_ values were calculated by means of GraphPad Prism 4 (GraphPad Software, San Diego, CA, USA).

### Determination of *in situ* caspase-8 activity

To analyse the effects of d-homoestrone on the activity of caspase-8, the enzyme involved in the extrinsic apoptotic pathway, a commercially available colourimetric assay was performed. Briefly, near-confluent HeLa cells were seeded in tissue culture flasks (10^6^ and 10^7^ cells/flask for untreated control and treated samples, respectively) and grown overnight under standard cell culturing conditions. The cells were then incubated with increasing concentrations (1.25, 2.5 and 5.0 μM) of the test compound for 72 hrs. Meanwhile, the medium of the untreated control cells was replaced. After incubation, the cells were counted, centrifuged and washed with PBS. Aliquots containing 10^7^ cells were suspended in 100 μl of kit lysis buffer and incubated on ice for 20 min. The lysed cells were subsequently centrifuged and the supernatants were used for the measurement. In accordance with the manufacturer’s protocol, 10 μl portions of treated and untreated supernatants were incubated with 10 μl of acetyl-Ile-Glu-Thr-Asp *p*-nitroaniline, a selective caspase-8 substrate, in a final volume of 100 μl in assay buffer. As a control experiment, 10 μl of each sample was incubated with 10 μl of caspase-8 substrate and 2.0 μl of acetyl-Ile-Glu-Thr-Asp-aldehyde, a selective caspase-8 inhibitor, in a final volume of 100 μl in assay buffer. Each sample was prepared in five parallels. A standard solution of caspase-8 was used as positive control in parallel with inhibitor-treated samples. After an overnight incubation at 37°C, the absorbance of the *p*-nitroaniline released was measured at 405 nm. Comparison of the absorbance of *p*-nitroaniline from the treated sample with that of an untreated control sample allowed determination of the fold increase in caspase-8 activity.

### Determination of *in situ* caspase-9 activity

To analyse the effects of d-homoestrone on the proteolytic activity of caspase-9, the enzyme involved in the intrinsic apoptotic pathway, a commercially available colourimetric assay (Invitrogen, Carlsbad, CA, USA) was performed. The preparation of the cells before cell lysis was identical with the method described for the determination of *in situ* caspase-8 activity. Aliquots containing 3 × 10^6^ cells were then suspended in 50 μl of kit lysis buffer and incubated on ice for 10 min. The lysed cells were subsequently centrifuged and the supernatants were used for the measurement. In accordance with the manufacturer’s protocol, 50 μl portions of treated and untreated supernatants were incubated with 5.0 μl of Leu-Glu-His-Asp-*p*-nitroaniline, a selective caspase-9 substrate, in a final volume of 105 μl in reaction buffer containing 10 mM dithiothreitol. Each sample was prepared in five parallels. After an overnight incubation at 37°C, the absorbance of the *p*-nitroaniline released was measured at 405 nm. Comparison of the absorbance of *p*-nitroaniline from the treated sample with that of an untreated control sample allowed determination of the fold increase in caspase-9 activity.

### Analysis of the G2/M phase transition by flow cytometry

For a quantitative characterization of the action of the test compound on the G2/M phase transition in HeLa cells, immunocytochemical flow cytometric analysis was performed. HeLa cells (10^5^/well) were seeded in 6-well plates and allowed to proliferate for 48 hrs. On the 3rd experimental day, the cells were treated with 20 μM d-homoestrone for 24 hrs, *i.e*. the concentration and incubation period used in the previously reported cell cycle analysis 14. With regard to previous results, 2-ME was administered at 5.0 μM for 24 hrs to demonstrate its direct effect on the G2/M phase transition 10. 5.0 nM paclitaxel was applied as a positive control for 20 hrs 17 to demonstrate the adaptability of the experimental method. The medium of the control cells was concomitantly replaced. After incubation, the cells were counted, washed with PBS, centrifuged and resuspended in the 1× wash buffer of the commercially available flow cytometric kit (Millipore Co., Billerica, MA, USA). Samples were fixed by adding fixation buffer and incubated for 20 min. at 4°C. After removal of the supernatant by centrifugation, cells were resuspended in ice-cold 1× permeabilization buffer and incubated for 5 min. at 4°C. The supernatant was removed by centrifugation and the samples were washed once by adding 1× assay buffer. The cells were first stained with anti-phospho-histone H3 (Ser10) antibody directly conjugated to Alexa Fluor 488 for 1 hr at 4°C in the dark. After an additional washing step with 1× assay buffer, the cells were incubated with freshly-prepared propidium iodide/RNase solution for 30 min. at room temperature in the dark. Samples were analysed on a Partec CyFlow instrument (Partec GmbH, Münster, Germany). In each analysis, 20,000 events were recorded, and the percentage of the cells in the M phase was determined by using Flowing Software 2.5 (Cell Imaging Core, Turku Centre for Biotechnology, Turku, Finland). The fraction stained with anti-phospho-histone H3 (Ser10) antibody was regarded as the cell population in the M phase 18. Each sample was prepared in three parallels and the experiment was repeated twice.

### RT-PCR studies

The effects of the tested compound on the mRNA expression pattern of cyclin-dependent kinase 1 (Cdk1), together with cyclin B1 and B2, and its regulator factors Cdc25B and Cdc25C, which play a crucial role in the transition from the G2 to the M phase of the cell cycle, were determined by RT-PCR in HeLa cells. After a 48-hrs incubation period, the medium containing the various test compounds was discarded and the total RNA was isolated from the cells (5 × 10^5^) through use of the TRIzol Reagent in accordance with the instructions of the manufacturer (Life Technologies) 19. The pellet was resuspended in 100 μl of DNase- and RNase-free distilled water. The RNA concentrations of the samples were determined from their absorbances at 260 nm. The RNA (0.5 μg) was mixed with DNase- and RNase-free distilled water and 20 μM oligo(dT)15 (Promega, Madison, WI, USA), in a final reaction volume of 10 μl, and was incubated at 70°C for 5 min. After the mixture had been cooled to 4°C, 20 U of RNase inhibitor (Fermentas™, Thermo Fisher Scientific Inc., Waltham, MA, USA), 20 U of MMLV reverse transcriptase (Promega), 200 μM dNTP (Life Technologies) in 50 mM Tris-HCl, pH 8.3, 75 mM KCl and 5 mM MgCl_2_ in a final reaction volume of 10 μl were added. The mixture was incubated at 37°C for 60 min. The PCR was carried out with 5 μl of cDNA, 12.5 μl of AccuStart GelTrack PCR SuperMix (Quanta Biosciences Inc., Gaithersburg, MD, USA), 2 μl of 20 pM sense and the antisense primer of Cdk1, cyclin B1, cyclin B2, Cdc25B, Cdc25C and 3.5 μl of DNase- and RNase-free distilled water. The primer sequences used to amplify Cdk1, cyclin B1 and B2, Cdc25B and Cdc25C were described by Shi *et al*. 20, Bellanger *et al*. 21, Takemasa *et al*. 22 and Lau *et al*. 23, respectively. Human glyceraldehyde 3-phosphate dehydrogenase primers were used as internal control in all samples (Table S1) 24. The PCR was performed with an ESCO SWIFT MAXI thermal cycler (Esco Technologies Inc, Philadelphia, PA, USA) and the products were separated on 2% agarose gels, stained with ethidium bromide and photographed under a UV transilluminator. Each sample was prepared in three parallels and the experiments were repeated twice. Semiquantitative analysis was performed by densitometric scanning of the gel with Kodak IMAGE STATION 2000R (Eastman Kodak Co., Rochester, NY, USA).

### Western blotting studies

To investigate the action of d-homoestrone on the function of CDK1, phosphorylated and total stathmin protein expression was determined by western blot analysis. HeLa cells were harvested in 60-mm dishes at a density of 2 × 10^5^ cells/ml and treated with d-homoestrone for 48 hrs. Whole-cell extracts were prepared by washing the cells with PBS and suspending them in lysis buffer (50 mM Tris, 5 mM EDTA, 150 mM NaCl, 1% NP-40, 0.5% deoxycholic acid, 1 mM sodium orthovanadate, 100 μg/ml phenylmethanesulfonylfluoride (PMSF) and protease inhibitors) 25. 10 μg of protein per well was subjected to electrophoresis on 4–12% NuPAGE Bis–Tris Gel in XCell SureLock Mini-Cell Units (Invitrogen). Proteins were transferred from gels to nitrocellulose membranes though use of the iBlot Gel Transfer System (Invitrogen). Antibody binding was detected with the WesternBreeze Chemiluminescent Western blot immunodetection kit (Invitrogen). The blots were incubated on a shaker with stathmin (Op18: rabbit polyclonal antibody raised against amino acids 1-149 representing full-length human protein), phosphorylated stathmin (p-Op18: rabbit polyclonal antibody raised against a short amino acid sequence containing phosphorylated Ser25 of human protein) and β-actin polyclonal antibody (Santa Cruz Biotechnology, Santa Cruz, CA, USA) 1:200 in the blocking buffer. Each sample was prepared in three parallels and the experiments were repeated twice. Semiquantitative analysis was performed by densitometric scanning of the blot with the Kodak IMAGE STATION 2000R (Eastman Kodak Co.).

### Tubulin polymerization assay

The direct effect of d-homoestrone on tubulin polymerization was tested with the Tubulin Polymerization Assay Kit (Cytoskeleton Inc., Denver, CO, USA) according to the manufacturer’s recommendations. Briefly, 10 μl of 250 or 500 μM test compound solution was subjected to a pre-warmed (37°C), UV-transparent microplate; 10 μl of 10 μM paclitaxel and 10 μl of general tubulin buffer served as positive and untreated control, respectively. 100 μl of 3.0 mg/ml tubulin in 80 mM PIPES pH 6.9, 2 mM MgCl_2_, 0.5 mM ethylene glycol tetraacetic acid (EGTA), 1 mM guanosine triphosphate (GTP), 10.2% glycerol was added to each sample, and the microplate was immediately placed into a pre-warmed (37°C) UV-spectrophotometer (SpectoStarNano, BMG Labtech, Ortenberg, Germany) to start the recording of the reaction. A 60-min. kinetic measurement protocol was applied for determination of the absorbance of the reaction solutions at 1-min. intervals at 340 nm. For evaluation of the experimental data, a tubulin polymerization curve was created by plotting optical density against time, and the maximum reaction rate (V_max_; Δabsorbance/min.) was calculated. The differences between the absorbances determined at two consecutive measuring timepoints were calculated, and the highest difference was taken as the V_max_ value of the tested compound in the tubulin polymerization reaction. Each sample was prepared in two parallels and the measurements were repeated twice.

### Statistical analysis

For statistical evaluation, the experimental data were in all cases analysed by one-way anova with the Neumann–Keuls post test, using GraphPad Prism version 4.01 for Windows (GraphPad Software, San Diego, CA, USA).

## Results

### Selective antiproliferative effect of d-homoestrone on HeLa cells

The proliferation-inhibiting effect of d-homoestrone was demonstrated not only on the HeLa (HPV-18-positive) cervical cancer cell line 14, but also on two other cervical cancer cell lines: SiHa (HPV-16-positive) and C33A (HPV-negative). d-Homoestrone did not give rise to effective inhibition of the proliferation of either SiHa nor C33A cells. The calculated IC_50_ values were from three separate experiments 30.7 ± 0.4 μM for SiHa and 32.4 ± 6.3 μM for C33A cells. The calculated IC_50_ value of d-homoestrone on HeLa cells was previously reported to be 5.5 μM 14.

### Involvement of the intrinsic apoptotic pathway in programmed cell death induced by d-homoestrone

**Figure 2:**
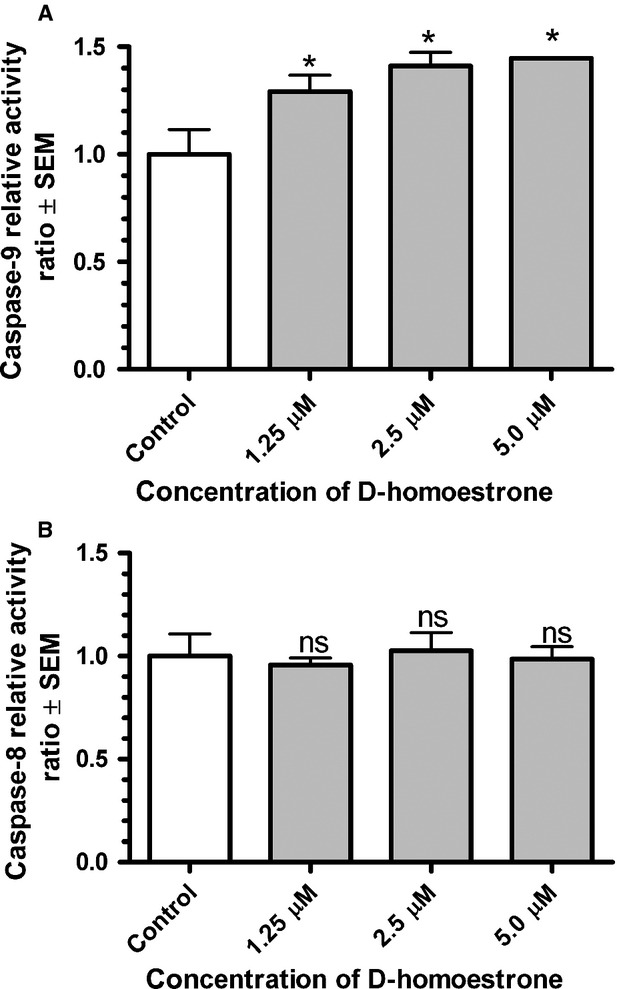
*In situ* measurement of caspase-9 (A) and -8 (B) activities in HeLa cells after treatment with d-homoestrone for 72 hrs. The activities of caspase-9 and -8 in d-homoestrone-treated samples are expressed as ratios relative to the activities of caspase-9 or -8 in the control (untreated) samples. Data are means ± SEM, *n* = 5. ns indicates *P* > 0.05, and * indicates *P* < 0.05 as compared with the untreated control samples.

To obtain novel and conclusive information about the mechanism of action of apoptosis induction triggered by d-homoestrone, the activities of caspase-8 and -9 on HeLa cells were determined after exposure to 1.25, 2.5 or 5.0 μM of the test compound for 72 hrs. d-Homoestrone induced a concentration-dependent and significant increase in *in situ* caspase-9 activity on HeLa cells relative to the untreated control samples, indicating the participation of the intrinsic pathway in the development of apoptotic cell death (Fig. 2A). However, no significant alteration in caspase-8 activity was detected in the d-homoestrone-treated HeLa cells as compared with the untreated control samples (Fig. 2B).

### d-Homoestrone blocks the cell cycle in the G2 phase

**Figure 3:**
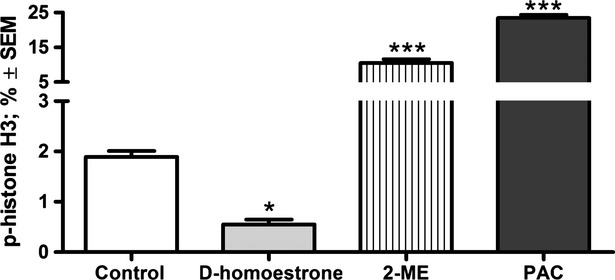
Effects of 20 μM d-homoestrone and 5.0 μM 2-ME on the expression of p-histone H3 protein in HeLa cells after incubation for 24 hrs, determined by flow cytometric analysis. Results are mean values ± SEM of the data on three separate measurements. * indicates *P* < 0.05, and *** indicates *P* < 0.001 as compared with the untreated control cells. PAC denotes 5.0 nM paclitaxel and 2-ME denotes 5.0 μM 2-methoxyestradiol.

d-Homoestrone has been revealed by flow cytometric analysis to increase the ratio of cells in the G2/M phase significantly 14. To establish its exact mechanism of action, immunocytochemical flow cytometric analysis was performed. The test compound significantly decreased the ratio of phosphorylated histone H3 protein relative to the untreated control HeLa cells (Fig. 3), indicating the reduction of the cells in the M phase. Paclitaxel, a widely known mitotic blocking agent 26, was used as positive control. It increased the ratio of phosphorylated histone H3 protein significantly as compared with the untreated control HeLa cells (Fig. 3). Moreover, after the treatment of HeLa cells with 2-ME for 24 hrs, the proportion of phosphorylated histone H3 protein was also significantly increased relative to the untreated control cells (Fig. 3).

### Inhibition of regulatory proteins of the G2/M phase transition at the mRNA level

**Figure 4:**
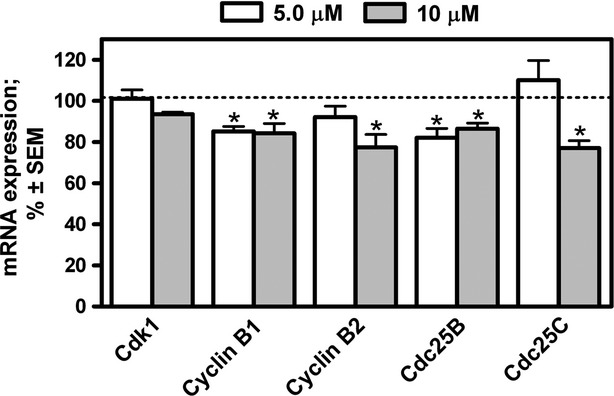
Effects of 5.0 μM (open bars) and 10 μM (grey bars) d-homoestrone on the mRNA expression of regulating factors participating in the G2/M transition in HeLa cells after incubation for 48 hrs, determined by reverse-transcription PCR. Results are mean values ± SEM of the data from two separate measurements, *n* = 6. * indicates *P* < 0.05 as compared with the untreated control samples (dashed line at 100%). Non-significant changes are not indicated. Cdk1 denotes cyclin-dependent kinase 1.

The G2/M transition is known to be a rigorously regulated process during the cell cycle 27. If the function of the regulatory pathway is affected, cells may not enter the M phase and accumulate in the G2 phase. Although the mRNA expression of the executioner enzyme (Cdk1) demonstrated a non-significant alteration in HeLa cells incubated with d-homoestrone for 48 hrs, those of the activating part of the Cdk1-cyclin B complex and its direct upstream regulatory factors (*e.g*. Cdc25B and Cdc25C) were significantly reduced relative to the untreated control cells. However, the mRNA expression of cyclin B2 and Cdc25C was reduced significantly in the presence of 10 μM d-homoestrone alone (Fig. 4).

### Functional blockade of the Cdk1-cyclin B complex

**Figure 5:**
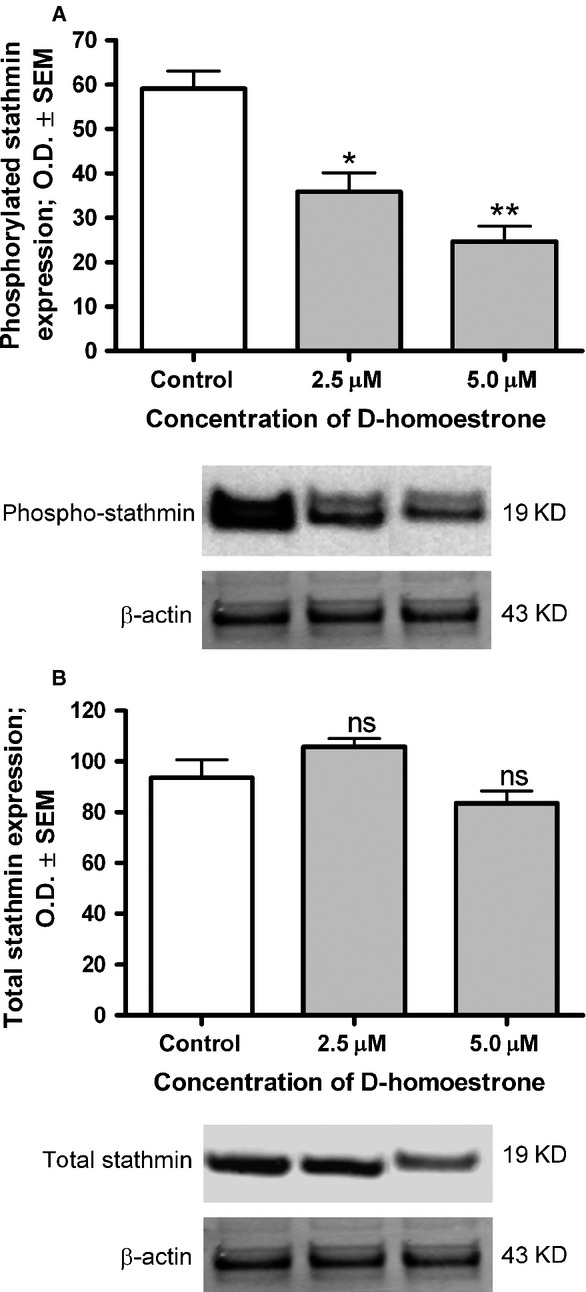
Effects of 2.5 and 5.0 μM d-homoestrone on the expression of phosphorylated (A) and total (B) stathmin protein in HeLa cells after incubation for 48 hrs, determined by western blot analysis. Results are mean values ± SEM of the data from two separate measurements, *n* = 6. ns indicates *P* > 0.05, * indicates *P* < 0.05 and ** indicates *P* < 0.01 as compared with the untreated control cells. The β-actin probe was used as internal control. Panels below are representative membrane pictures.

In response to a 48-hrs incubation with d-homoestrone, the protein expression of phospho-stathmin, a microtubule destabilizing protein phosphorylated and therefore inactivated by Cdk1, significantly decreased in comparison with the untreated control cells (Fig. 5A). However, the total protein expression of stathmin did not display a significant alteration as compared with the untreated control cells (Fig. 5B).

### d-Homoestrone does not elicit a direct effect on tubulin polymerization

**Figure 6:**
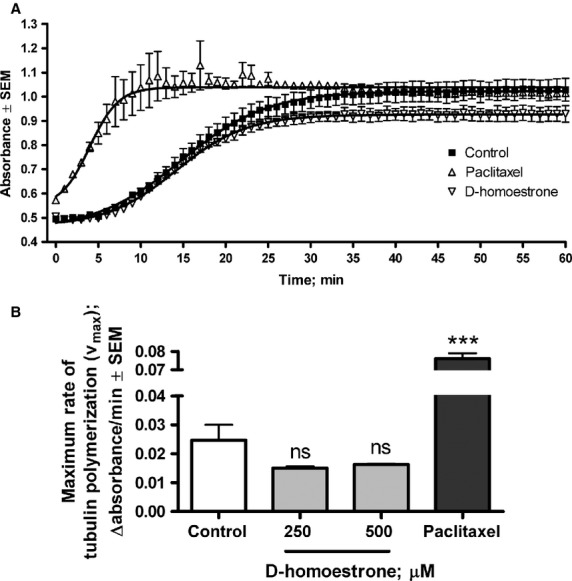
A representative kinetic curve of the effects of 250 μM d-homoestrone and 10 μM paclitaxel on tubulin polymerization (A). Direct effects of 250 and 500 μM d-homoestrone and 10 μM paclitaxel on the maximum rate of tubulin polymerization determined by an *in vitro* kinetic assay (B). Results are mean values ± SEM of the data from two separate measurements, *n* = 4. ns indicates *P* > 0.05 and *** indicates *P* < 0.001 as compared with the untreated control samples.

2-ME and other steroidal antiproliferative agents were earlier reported to affect the tubulin-microtubules system 28,29, and the effect of d-homoes trone on tubulin polymerization was therefore also determined. d-Homoestrone applied in two different concentrations (250 and 500 μM) did not significantly alter the maximum rate of tubulin polymerization relative to the untreated control samples in an *in vitro* 1-hr kinetic assay (Fig. 6A). In contrast, the positive control paclitaxel evoked a nearly threefold increase in V_max_ (Fig. 6B).

## Discussion

There have been numerous investigations of the antiproliferative mechanism of action of 2-ME, the first A-ring-modified estradiol derivative with promising anticancer activity. In view of the chemical similarity of the structures, the cancer cell proliferation-inhibiting mechanism of action of 2-ME might be regarded as a common mode of pharmacological action for all related antiproliferative estradiol derivatives. However, the present paper provides convincing data of an alternative mechanism of action of d-homoestrone, a 2-ME analogue with d-ring expansion. d-Homoestrone was earlier shown to exert a pronounced antiproliferative effect on HeLa cells by inducing apoptosis, preceded by cell cycle blockade at the G2/M phase 14. Our research into its detailed mechanism of anticancer action has revealed some intriguing differences from that in the case of 2-ME.

Antiproliferative assays have been performed with d-homoestrone on cervical cancer cell lines with various pathological backgrounds (HPV-16-positive SiHa and HPV-negative C33A cell lines) compared to HPV-18-positive HeLa cells. The present and previous results reveal that the anticancer effect of d-homoestrone is selective for HeLa cells. Although the expressions of the oncoproteins E6 and E7 are detected in both HPV-positive cervical cell lines, which deregulate fundamental cellular events such as apoptosis, cell cycle, DNA repair, senescence and differentiation [reviewed in 30], a slight difference in the apoptotic machinery of these cell lines can be observed. Aréchaga-Ocampo *et al*. 31 reported different expression profiles of caspases in different HPV-positive cervical cell lines. In HeLa cells (HPV-18+), caspase-3 and -6 displayed low levels of expression, whereas caspase-7, -8 and -9 were expressed at high levels. In contrast, most of the caspases were highly expressed in SiHa cells (HPV-16+), the exception was caspase-8. This difference might be a possible explanation of the selective susceptibility of HeLa cells to d-homoestrone.

2-Methoxyestradiol has been reported to induce the process of programmed cell death in numerous cancer cell lines, including HeLa(S3) cells, as demonstrated by flow cytometry, fluorescent microscopy and DNA fragmentation data 10. During the investigation of the mechanism of apoptosis, other research groups reported that 2-ME is able to activate both the intrinsic 11 and the extrinsic 32 apoptotic pathway in HeLa cells. The present results on caspase-8 and -9 activity allow the conclusion that the previously demonstrated apoptosis-inducing effect of d-homoestrone develops only through the activation of the intrinsic pathway, since the activity of caspase-8 was not altered significantly in HeLa cells after d-homoestrone treatment.

Earlier cell cycle analyses on various cancer cell lines (including HeLa cells) proved that the proportion of cells in the G2/M phase increased significantly on exposure to 2-ME 10,33–35. Similarly, 5.0 μM d-homoestrone evoked a G2/M phase blockade of HeLa cells 14. After Li *et al*. 10 had established that the G2/M phase arrest induced by 2-ME was mainly due to the inhibition of mitosis at the metaphase, the question arose of whether the cell cycle blockade induced by d-homoestrone develops in the G2 or the M phase. It was earlier reported that an important detectable ‘label’ on the chromosomes indicates to the cell that duplicated chromosomes are ready for segregation. The amount of this molecule, Ser10 phosphorylated histone H3, reaches its maximum in the metaphase 36. Moreover, the presence of phosphorylated histone H3 is a prerequisite for chromatin condensation, which is regarded as a sign of the onset of the prophase. In our present investigation, the analysis of the presence of phosphorylated histone H3 on Ser10 revealed that the proportion of d-homoestrone-treated HeLa cells containing this form of the protein was decreased dramatically as compared with the control cells, suggesting that these cells are neither able to arrive at the metaphase of cell cycle nor to demonstrate the initiation of the first step of mitosis. Additionally, 2-ME significantly increased the proportion of phosphorylated histone H3 as protein compared with that in untreated control samples, indicating an increase in the HeLa cells in the M phase. This result is in good agreement with the recently published data on the cell cycle blockade induced by 2-ME. In morphological assays, 2-ME-induced, abnormal spindle formation was observed in the metaphase in HeLa(S3) cells 10. Furthermore, Choi and Zhu 35 reported that 2-ME selectively induced a mitotic prometaphase arrest due to its inhibitory effect on microtubule formation, followed by activation of the cyclin B1-CDK1 complex, mediated in part by c-Jun N-terminal kinase (JNK) and mitotic arrest deficient 2 (MAD2) proteins. These experimental findings allow the conclusion that 2-ME exerts a cell cycle blockade at the beginning of mitosis and not in the G2 phase. This difference between the effects of 2-ME and d-homoestrone at this step of the cell proliferation process is a crucial one.

The cell cycle is a strictly regulated process which ensures the development of two completely identical and viable cells. It is divided into specific phases according to unique intracellular events, and the transition from one phase to the following one is governed by cascade mechanisms involving various regulatory proteins (*e.g*. phosphatases, kinases, *etc*.). The main executor protein of the G2/M phase transition is Cdk1, the functional activity of which is strongly dependent on the expression of cyclin B proteins 27. During physiological functioning, this complex phosphorylates the proteins responsible for mitotic entry and progression. The effects of 2-ME on Cdk1 expression and activity and on cyclin B1 expression have been widely investigated in numerous cancer cell lines, but not in HeLa cells. The opposite actions of 2-ME on Cdk1 and cyclin B1 expression have been demonstrated, depending on the cell type investigated. Gong *et al*. 34 observed an inhibitory effect of 2-ME on Cdk1-cyclin B1 activity, followed by a cell cycle arrest at G2/M in RL95-2 human endometrial cells. Two possible molecular mechanisms have been proposed to explain the cell cycle blockade; the direct activation of p53 at Ser15, and the indirect activation of p53 at Ser20 *via* the induction of checkpoint kinase 1. In both cases, upregulation of the downstream p21 results in a decreased activity of Cdk1-cyclin B1. Similarly, a decrease in the expression of Cdk1 and cyclin B1 protein was demonstrated in MCF-7 cells as a result of 2-ME treatment 37. However, two other research groups reported that 2-ME treatment leads to an increased level of Cdk1 and cyclin B1 protein or only of cyclin B1 protein in MCF-7 cells 33,35. However, it was finally concluded that the observed effect may be connected with mitotic progression and not with mitotic entry. In spite of the limitations of the applied PCR technique, d-homoestrone with a selective antiproliferative effect on HeLa cells proved to decrease the mRNA expressions of cyclin B1 and B2 significantly, but not to affect that of Cdk1, suggesting a reduced functional activity of the complex during the prophase of mitotic entry. The reduced functional activity of the Cdk1-cyclin B complex was confirmed by investigation of the phosphorylated form and the total stathmin expression. Stathmin is one of the several targets of Cdk1; the phosphorylated form of this protein becomes inactive, allowing the formation of microtubules from tubulin heterodimers sequestered earlier by active stathmin, or the inhibition of its microtubule catastrophe-promoting activity when cells enter mitosis (*i.e*. in the prophase) 38. Our results revealed a significantly decreased protein expression of phosphorylated (and therefore inactivated) stathmin after a 48-hrs d-homoestrone treatment, whereas the total stathim expression did not exhibit a marked change. This suggests that d-homoestrone induces the expression of active stathmin, resulting in dysregulation of the dynamic instability of the tubulin-microtubule system.

On the other hand, there is an upstream regulatory system which is able to influence the activity of the Cdk1-cyclin B complex. The members of this system include Cdc25B and Cdc25C phosphatases, which activate Cdk1 by dephosphorylating Tyr15 and Thr14 39. Moreover, Cdc25A and B are known to be overexpressed in a diverse array of human cancers, suggesting an important role of the Cdc25s in the development of uncontrolled cancer cell division 40. In our experiments, the Cdc25B mRNA expression was demonstrated to be reduced significantly after a 48-hr treatment with 5.0 or 10 μM d-homoestrone, suggesting decreasing enzymatic activity. However, the change in the mRNA level of Cdc25C, the other phosphatase participating in the control of entry into mitosis, decreased significantly only after treatment with the higher dose of d-homoestrone. These findings are consistent with previous results indicating that Cdc25B is the initial trigger of mitotic onset and its expression starts slightly earlier than that of Cdc25C 41. Until recently, the effects of 2-ME on the activity and/or expression of Cdc25 phosphatases had been only poorly investigated. In a cell-free experiment, it inhibited the activity of Cdc25B and C directly 42, and it was also shown to alter the expression profile of the active or inactive form of Cdc25C in different cancer cell lines 35,43,44.

Our investigation aimed at an understanding of the antiproliferative mechanism of action of d-homoestrone, additionally demonstrated its direct effect on tubulin polymerization. 2-ME has been reported to inhibit tubulin polymerization in consequence of its interaction with the colchicine-binding site of β-tubulin 12. This effect seems to develop during the initial steps of mitosis, which it may allow the conclusion that 2-ME causes the cell cycle arrest at the M phase. In contrast, d-homoestrone did not exhibit any significant action on tubulin polymerization relative to non-treated control samples in an *in vitro*, cell-free experimental setting. This difference is another significant factor in the comparison of the mechanism of antiproliferative action of d-homoestrone with that of 2-ME.

Overall, our current results suggest that d-homoestrone causes a functional loss of the Cdk1-cyclin B complex, resulting in failure of the G2/M transition in HeLa cells. In contrast with 2-ME, this molecule does not interfere directly with the dynamic equilibrium of tubulin-microtubule system, but because of the inhibited inactivation of stathmin it indirectly disturbs the microtubule formation at the beginning of mitotic entry. Finally, our additional experimental data indicate that d-homoestrone arrests the cell cycle progression immediately before the HeLa cells start to enter mitosis; this is followed by the induction of programmed cell death which, unlike the case with 2-ME, is executed solely *via* the intrinsic apoptotic pathway. We consider that this alternative mechanism of anticancer action of d-homoestrone in HeLa cells is a promising novel feature in the search for lead compounds against human cervical carcinoma.

## Supporting Information

**Table S1.** Primers and PCR conditions of cell cycle regulator genes, the GeneBank access numbers and the length of PCR products.
