# Supplementary material for: A molecular understanding of d-homoestrone-induced G2/M cell cycle arrest in HeLa human cervical carcinoma cells
Source: J Cell Mol Med. 2015 Jul 31;19(10):2365–74. doi: 10.1111/jcmm.12587 (PMC4594678; doi:10.1111/jcmm.12587)
Supplement: Supplementary file 1 [file jcmm0019-2365-sd1.docx]

**Supplementary table 1**

Primers and PCR conditions of cell cycle regulator genes, the Genebank access numbers and the length of PCR products

| **Name:** | **Primer sequence** | **Gene ID** | **Product size**  **(bp)** | **Coupling temp.**  **(°C)** |
| --- | --- | --- | --- | --- |
| Cdk1 | F: ACTGGCTGATTTTGGCCTTGCC  R: TGAGTAACGAGCTGACCCCAGCAA | 983 | 118 | 62 |
| cyclinB1 | F: AATAAGGAGGGAGCAGTGCG  R: GAAGAGCCAGCCTAGCCTCAG | 891 | 51 | 60 |
| cyclinB2 | F: GCGTTGGCATTATGGATCG  R: TCTTCCGGGAAACTGGCTG | 9133 | 51 | 60 |
| Cdc25B | F: CACGCCCGTGCAGAATAAGC  R: ATGACTCTCTTGTCCAGGCTACAGG | 994 | 417 | 60 |
| Cdc25C | F: TTTTTCCAAGGTATGTGCGCTG  R: TGGAACTTCCCCGACAGTAAGG | 995 | 102 | 56 |
| hGAPDH | F: ACCCAGAAGACTGTGGATGG  R: TGCTGTAGCCAAATTCGTTG | 2597 | 415 | 55 |
